# EFFECTIVENESS OF THE LIGATION OF INTERSPHINCTERIC FISTULA TRACT (LIFT) IN THE TREATMENT OF ANAL FISTULA: INITIAL RESULTS

**DOI:** 10.1590/S0102-67202014000200004

**Published:** 2014

**Authors:** Sergio Danilo Tanahara TOMIYOSHI, Carlos Henrique Marques DOS SANTOS

**Affiliations:** Hospital Regional de Mato Grosso do Sul, Campo Grande, MS, Brazil

**Keywords:** Anal fistula, Fecal incontinence, Surgery

## Abstract

**Background:**

The abscesses and anal fistulas represent about 70% of perianal suppuration, with an estimated incidence of 1/10000 inhabitants per year and representing 5% of queries in coloproctology.

**Aim:**

To evaluate the effectiveness of the interesphincteric ligation technique of the fistulous tract in the treatment of anal fistula.

**Methods:**

The records of eight patients who underwent this technique, evaluating age, gender and presence of incontinence were studied. Was named technical first-step the passage of cotton thread to promote the correct individualization of the fistula and, as the second, the surgical procedure.

**Results:**

Two patients were men and eight women. The mean age was 42.8 years. Of these, seven (87.5%) had complete healing of the fistula; six were cured only with this procedure and one required additional operation with simple fistulotomy. Only one patient developed fecal incontinence which was documented by anorectal manometry. There were no deaths in this series.

**Conclusion:**

The interesphincteric ligation technique of the fistulous tract proved to be effective for the treatment of anal fistula and should not be discouraged despite the occurrence of eventual fecal incontinence.

## Abstract (pt)

**Racional:**

Os abscessos e fístulas anais representam cerca de 70% do total das supurações perianais, estimando-se incidência de 1/10000 habitantes por ano e representando 5% das consultas em coloproctologia.

**Objetivo:**

Avaliar a eficácia da técnica de ligadura interesfincteriana do trato fistuloso no tratamento de fístula anal.

**Métodos:**

Foram estudados os prontuários de oito pacientes submetidos à essa técnica avaliando-se idade, sexo e presença de incontinência. Denominou–se primeiro tempo da técnica a passagem de sedenho com fio de algodão zero a fim da correta individualização do trajeto fistuloso e, como segundo tempo a realização do procedimento.

**Resultados:**

Dois pacientes eram homens e oito mulheres. A média de idade foi de 42,8 anos. Desse total, sete (87,5%) evoluíram com cura completa da fístula, seis somente com esse procedimento e um necessitou passar por operação adicional após a realização do segundo tempo, com fistulotomia simples. Apenas uma paciente evoluiu com incontinência fecal a qual foi documentada por exame de manometria anorretal. Não houve óbitos nessa série.

**Conclusão:**

A técnica de ligadura interesfincteriana do trato fistuloso mostrou–se eficaz para o tratamento da fístula anal e não deve ser desestimulada a despeito da ocorrência de incontinência fecal.

## INTRODUCTION

Abscesses and anal fistulas represent about 70% of perianal suppuration, with an estimated incidence of 1/10,000 inhabitants per year and representing 5% of queries in coloproctology^1^.

Anal fistula is the chronic phase of anorectal infection is characterized by chronic purulent drainage or cyclic pain associated with acute relapse of the abscess followed by intermittent spontaneous decompression^2^. About 65% of patients with perianal abscess will develop chronic or recurrent anal fistula. The operation for correction of anal fistula aims its cure with preservation of continence mechanism.

Among the current treatment options are: fistulotomy, application of fibrin glue, endorectal advancement flap, VAAFT (video-assisted technique) and ligation of the intersphincteric fistula tract (LIFT)^3^ .

The cure rate of fistulotomy is 0-64%, and up to 17% incontinence^3^. Use of fibrin glue cure up to 60% and does not cause incontinence^3^. Endorectal advancement has a cure rate of 98% with incontinence of up to 35%^3^. Following a year, VAAFT obtains cure of about 85% without incontinence^4^

In 2007 Arun Rojanasakul et al. Department of Colorectal Surgery, Chulalongkorn University, Bangok, Thailand, developed the technique Ligation of the Intersphincteric Fistula Tract (LIFT)^5^. The central idea of this procedure is that the excision and ligation of intersphincteric tract can occlude the entry of faecal particles in the fistula and, at the same time, eliminate the septic focus intersphincteric. This could result in the cure of anal fistula. This procedure aims to maintain the anal sphincter intact, preserving continence postoperatively^3,5^.

The treatment of anal fistula is mainly surgical to eliminate the fistula, prevent recurrence and preserve anal continence. However, among the various alternatives for the treatment of anal fistulas, until the moment, none of them is considered as the technique of choice due to their recurrence rates and incontinence.

Therefore, as there is no rigid model of choice of surgical treatment to be used, the current trend is that the techniques with preservation of the anal sphincter, as the LIFT and VAAFT, gain more space in the treatment of anal fistulas.

The objective of this study is to present the initial results in medium term the use of LIFT in the treatment of these fistulas.

## METHOD

The study was approved by the Ethics Committee in Research of the Hospital Regional de Mato Grosso do Sul, Campo Grande, MS, Brazil. All patients were informed by the surgical team about the technique to be used, expected outcome and complications. The study was prospective.

The group included in this study was composed of men and women suffering from perianal fistulas cryptoglandular transphincteric without previous surgery, aged 21-68 years belonging to the service of coloproctology of Hospital Regional de Mato Grosso do Sul, in the period from March 2011 to July 2013. Patients with perianal fistulas from another source, Crohn's disease, tuberculosis, cancer and recurrent anal fistulas as well as those that were previously operated by another surgeon were excluded.

All patients were underwent two surgical steps.

The first step, after spinal anesthesia with morphine, and with the patient in the Lloyd Daves position, held intraoperative verification of the fistula, by passing through the external hole a stylus to check it in the inner hole. In all cases, the fistula was characterized as transphincteric. Once defined the path, was realized the first operative step which consisted in passing wire seton (five strands of cotton thread) that were tied together with manual surgical knots.

**Figure 1:**
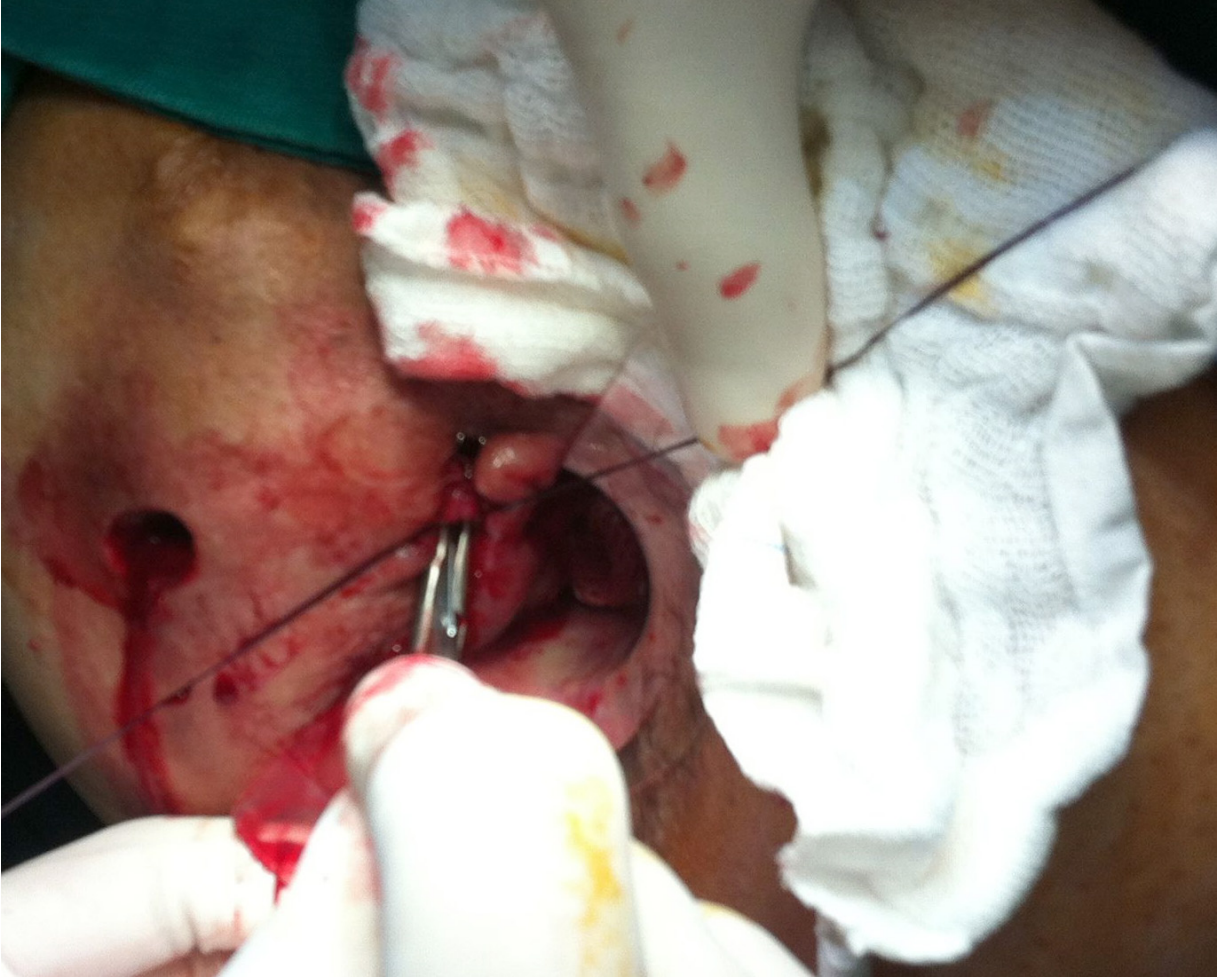
The yarns are repairing the fistula tract: the right one next to the edge of the internal sphincter and the left is by the external sphincter

The second step was between 4-6 weeks after the first. The position of the patient and anesthetic technique were equal. Before removing the seton, new exploration of the fistula was performed with stylus. There was no cases of emergence of a "new path" in this period. Proceeding with the removal of seton started the LIFT technique . From the path palpated after removal of seton, was that of a transverse incision medial to the external orifice of the fistula allowing meticulous dissection with scissors and electrocautery. Exposing the intersphincteric plane was facilitated using surgical retractors type Farabeuf. The intersphincteric path was ligated at two points: one on the emergence of the internal sphincter, and other on the external sphincter with polyglactin 2.0. After that, the remaining intersphincteric tract was resected in order to prevent infected tissue remained in the wound. The path of the fistula was again curetted and confirmed the effectiveness of ligation with no penetration of the anal canal curette by occlusion of the fistula point of emergence of the internal sphincter muscle. The external hole was left open to heal by secondary intention and also that this period could promote good drainage of the surgical wound (Figures 1 e 2).

**Figure 2:**
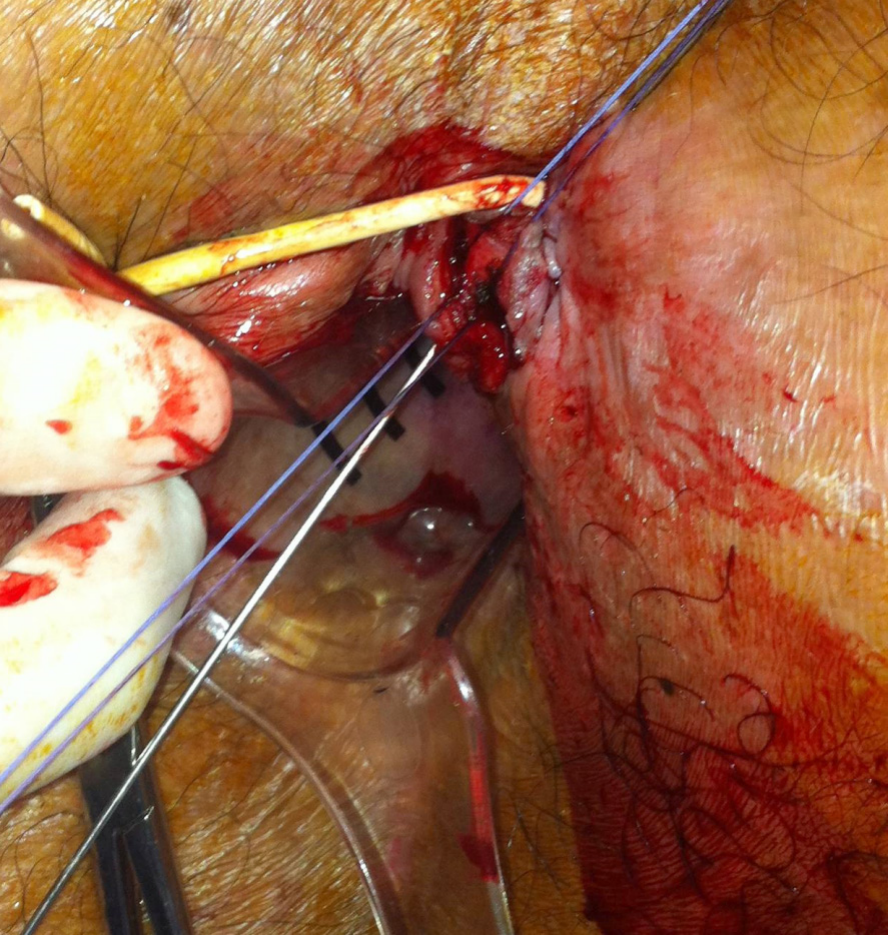
The second step of the technique, with correct identification of the fistula tract

After hospital discharge, patients were invited to attend to first follow-up visit 15 days after the initial procedure. The second consultation was on the 45^th^ day. It was considered cured patients who denied leaking stool by wound. Those in the second query still had symptoms of anal fistula were instructed to maintain the basic care of postoperative proctologic operations and invited to consult within 30 days.

It was considered that the recurrence after the third query still had symptoms of anal fistula. For these cases, it was proposed new surgical procedure may or may not be LIFT again. The presence of perianal abscess in any query postoperative was considered as complication and its treatment was recommended the use of quinolone for 14 days and subsequently revalued by the surgical team.

Fecal incontinence was considered in patients who were continent before the operation, but had postoperatively obvious injury of sphincter function. Patients who were discharged were instructed to return the query in case of reappearance of symptoms.

## RESULTS

In the analyzed period, eight patients were referred for surgical treatment by LIFT technique. In outpatient scheduled for 15 days after discharge, all evolved satisfactorily without any need for early re-intervention for drainage of abscess.

In the new review, 45 days after the first visit, six patients were considered cured; only one had leakage of fecal contents through the orifice of the fistula; another complained of incontinence.

For the patient with leakage through the opening of the fistula was scheduled to be consulted within 30 days and due to the permanence of the complaint, was opted for surgical intervention with fistulotomy with obtaining the cure of the condition.

For the patient with fecal incontinence was requested rectal manometry that confirm this diagnosis.

## DISCUSSION 

The initial study describing the technique^3^ was composed of 17 patients and primary cure rate was 94.4%; one patient underwent reoperation for the same technique LIFT obtaining cure after. There was no description of incontinence in this study.

Huda e Ashok^6^ reviewed the initial publication in order to establish more rigid inclusion criteria to identify patients who would benefit from the operation for fistula repair by LIFT technique and achieved 100% success in fistula closure after the first procedure and no patient had loss of continence.

Sileri et al^7^, in a prospective study of 18 patients achieved a cure rate of 83% with only three recurrences - the complementary treatment was fistulotomy in one patient and two other endorectal advancement - with subsequent complete healing of the fistula. There were also no cases of incontinence in this study.

Makhlouf and Korany^8^ in a series of 30 patients (25 men), mean age of 36.5 years who underwent LIFT showed complete cure rate of 90%; one patient with abscess six months after the initial procedure and three with recurrence. There were no cases of incontinence.

It is clear that LIFT has results that prove its effectiveness. The articles cited are consistent with this publication regarding the positive outcome of the technique, which stimulates to use LIFT when needed. Perhaps the key to that cure rates reach 100% in the first intervention, is the strict selection of patients in whom the characteristics of the fistulas are favorable to the use of this technique.

Due to the benefits mentioned, the LIFT technique has assumed a good surgical space and should remain with considerable one in relation to the various treatment options for anal fistula. It is expected that further publications with larger sample size to confirm the effectiveness of LIFT encouraging, more and more surgeons to use this technique.

## CONCLUSION

The Ligation of Intersphincteric Fistula Tract (LIFT) technique in mid-term evaluation is effective for the treatment of anal fistula.
